# Latent *Neoehrlichia mikurensis* Infections May Be Reactivated in Patients With B‐Cell Lymphomas Treated With Rituximab

**DOI:** 10.1111/imm.70120

**Published:** 2026-02-05

**Authors:** Linda Wass, Catharina Lewerin, Daniel Jaén‐Luchoro, Christine Lingblom, Christine Wennerås

**Affiliations:** ^1^ Department of Clinical Microbiology Sahlgrenska University Hospital Gothenburg Sweden; ^2^ Department of Infectious Diseases, The Sahlgrenska Academy University of Gothenburg Gothenburg Sweden; ^3^ Department of Hematology and Coagulation Sahlgrenska University Hospital Gothenburg Sweden; ^4^ Department of Internal Medicine and Clinical Nutrition, The Sahlgrenska Academy University of Gothenburg Gothenburg Sweden

**Keywords:** B‐cell lymphoma, CXCL10, gamma delta T cells, interferon‐gamma, *Neoehrlichia mikurensis*

## Abstract

The intracellular, tick‐borne bacterium *Neoehrlichia (N.) mikurensis* can cause neoehrlichiosis in patients with compromised B‐cell defences, while immunocompetent individuals are frequently healthy carriers of the infection. We hypothesised that *N. mikurensis* induces latent infections that reactivate when B‐cell immunity is compromised. We tested this hypothesis by determining the incidence of *N. mikurensis* reactivation in 97 patients with B‐cell lymphomas who were treated with anti‐CD20 antibody therapy (rituximab) and evaluating the presence of *N. mikurensis*‐specific T cells in latently infected individuals. Four patients (4%) reactivated *N. mikurensis* infection and four patients (4%) had asymptomatic infection before the initiation of B‐cell suppression. All eight patients who were infected with *N. mikurensis* had *N. mikurensis*‐specific, perforin‐expressing Th1 and CD8+ T‐cell populations with up‐regulation of CXCL10 and IFN‐γ, in contrast to the noninfected lymphoma patients who lacked these T‐cell subsets. The infected lymphoma patients also had expanded γδ T‐cell populations. This study supports the notion of latent, reactivatable *N. mikurensis* infections.

## Graphical abstract

Eight percent of this cohort of patients had a latent *Neoehrlichia mikurensis* infection. It was only the latently infected patients who had *N. mikurensis*‐specific T cells, not the matched B‐cell lymphoma patients without the infection. The T‐cell responses of latently infected patients included perforin‐expressing Th1 and CD8+ T cells that upregulated interferon‐gamma and CXCL10 upon stimulation with *N. mikurensis*‐derived peptides, as well as tenfold larger populations of gamma delta T cells compared with noninfected lymphoma control patients.

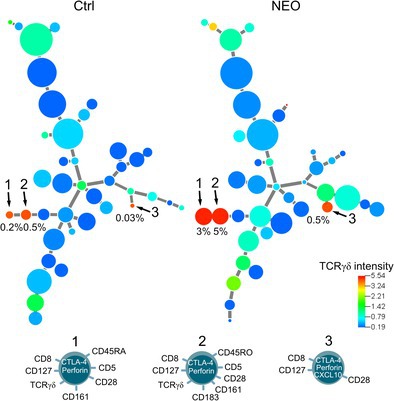

## 1 Introduction

The first case reports of human infections caused by the tick‐borne bacterium *Neoehrlichia mikurensis* were published in 2010 from Sweden, Germany and Switzerland [1, 2, 3]. This bacterial species belongs to the family *Anaplasmataceae*, along with other species that are pathogenic for humans, such as *Anaplasma phagocytophilum* and *Ehrlichia chaffeensis* [4]. Although *N. mikurensis* is widespread among ticks and rodents in Europe and northern Asia, it has not yet been discovered on the American continent [5]. This emerging pathogen causes the infectious disease neoehrlichiosis, which typically involves fever with night sweats, impaired general condition and localised pain [6]. More than half of the patients with neoehrlichiosis also present with unexplained vascular and thromboembolic events [7], which are probably attributable to the fact that *N. mikurensis* has tropism for vascular endothelial cells [8]. Since these intracellular bacteria do not grow in cell‐free blood culture media and no serologic tests are available at present, the only diagnostic option currently is PCR testing of blood samples [5]. The novelty of this agent, together with the limited diagnostic alternatives, is believed to contribute to this infection frequently being missed and misunderstood in the clinical setting.

The main mode of transmission of *N. mikurensis* infection is tick bites [5], although blood transfusions have also been implicated [9]. The incubation period of neoehrlichiosis is uncertain, and several studies have suggested that long‐term asymptomatic carriage of *N. mikurensis* can occur [10, 11, 12, 13], with the longest reported suspected carriage being in excess of 1 year [9]. A caveat is that none of these studies have excluded the possibility of repeated tick‐mediated infections with *N. mikurensis*. B cells are a crucial part of the infectious defence against *N. mikurensis*, as evidenced by the severity of neoehrlichiosis in patients with compromised B‐cell immunity, for example, patients with malignant B‐cell lymphomas, rheumatologic or other autoimmune diseases who are treated with B‐cell suppressive medications such as rituximab, which is a monoclonal antibody that targets CD20 on B cells [5, 14, 15].

The aim of this study was to investigate whether *N. mikurensis* causes latent infections that can be reactivated when B‐cell defences are compromised. To this end, we monitored the rate of *N. mikurensis* reactivation in patients with malignant B‐cell lymphomas living in a *N. mikurensis*‐endemic area (western Sweden) who were scheduled to start treatment with rituximab‐containing antilymphoma regimens, typically administered over a period of 4–6 months. The patients were tested for *N. mikurensis* infection by PCR before, mid‐way through and after termination of the rituximab regimen. We hypothesised that patients who exhibited reactivation of the infection would have *N. mikurensis*‐specific T cells in their blood prior to the start of rituximab therapy and that patients who remained infection‐free throughout the study period would lack *N. mikurensis*‐specific T cells. We also measured the interferon‐gamma (IFN‐γ) and CXCL10 responses to *N. mikurensis* in these patients, inspired by the IFN‐γ release assays used to diagnose latent tuberculosis (TB) and based on our own findings that these cytokines are present at increased levels in the blood samples of immuno‐suppressed patients with neoehrlichiosis [16]. *N. mikurensis*‐specific T‐cell responses were determined by evaluating the capacity of T cells to proliferate and identifying the types of T‐cell subsets that become activated upon in vitro stimulation with peptides derived from an outer membrane protein of *N. mikurensis* that belongs to the P44/Msp2 family of proteins. The P44/Msp2 protein used appears to be highly specific for *N. mikurensis*; the most closely related P44/Msp2 homologue has only 50% amino acid sequence identity, and is harboured by *Candidatus* Neoehrlichia lotoris, a species that is found in raccoons but is not known to infect humans [17]. Moreover, P44/Msp2 proteins have been shown to elicit immune responses in humans to related bacterial species, for example, *A. phagocytophilum* [18].

## 2 Results

*Incidence of N. mikurensis reactivation in lymphoma patients treated with rituximab*. The study group comprised 97 patients, two‐thirds of whom were men (68%), and the median age was 69 years. The patients were afflicted by 17 different types of malignant B‐cell lymphomas, with the most common being diffuse large B‐cell lymphoma (Table 1). Four percent (4/97) of the patients tested positive by *N. mikurensis* PCR after the start of rituximab therapy, two mid‐way through the treatment (Days 79 and 105 after the start of rituximab), and two after completion of the treatment (Days 215 and 222). An additional four patients (4%) were *N. mikurensis* PCR‐positive already at the start of the study, before having received rituximab treatment. None of the eight patients was aware of having an infection, nor did any of them display symptoms or clinical signs of *N. mikurensis* infection. The eight *N. mikurensis* PCR test results of the infected patients had relatively high cycle threshold (CT) values (median, 36; min–max, 27–39), indicative of low concentrations of bacteria in the blood. All the patients who tested positive for *N. mikurensis* were treated with oral doxycycline for 3 weeks and cleared the infection, as verified by PCR.

**TABLE 1:** Clinical characteristics of the study participants (*n* = 97).

Patients	*N*	%
Median age (min–max)	69 (44–92)	
Male	66	68
Type of B‐cell lymphoma		
Diffuse large B‐cell lymphoma	26	27
Mantle cell lymphoma	20^a^	21
Follicular lymphoma	13^b^	13
Grade 1	3	
Grade 2	4	
Grade 3a	6	
Chronic lymphocytic leukaemia	11	11
Waldenstrom macroglobulinaemia	7^a^	7.2
Extra‐nodal marginal zone lymphoma	5	5.2
Splenic marginal zone lymphoma	4	4.1
Indolent unclassified splenic lymphoma	3	3.1
Hodgkin's lymphoma	2^b^	2.1
Hairy cell leukaemia	1	1.0
Small lymphocytic lymphoma	1	1.0
Other B‐cell lymphomas	5	5.2

^a^ One patient had mantle cell lymphoma and Waldenstrom macroglobulinaemia.

^b^ One patient had follicular lymphoma and Hodgkin's lymphoma.

**FIGURE 1:**
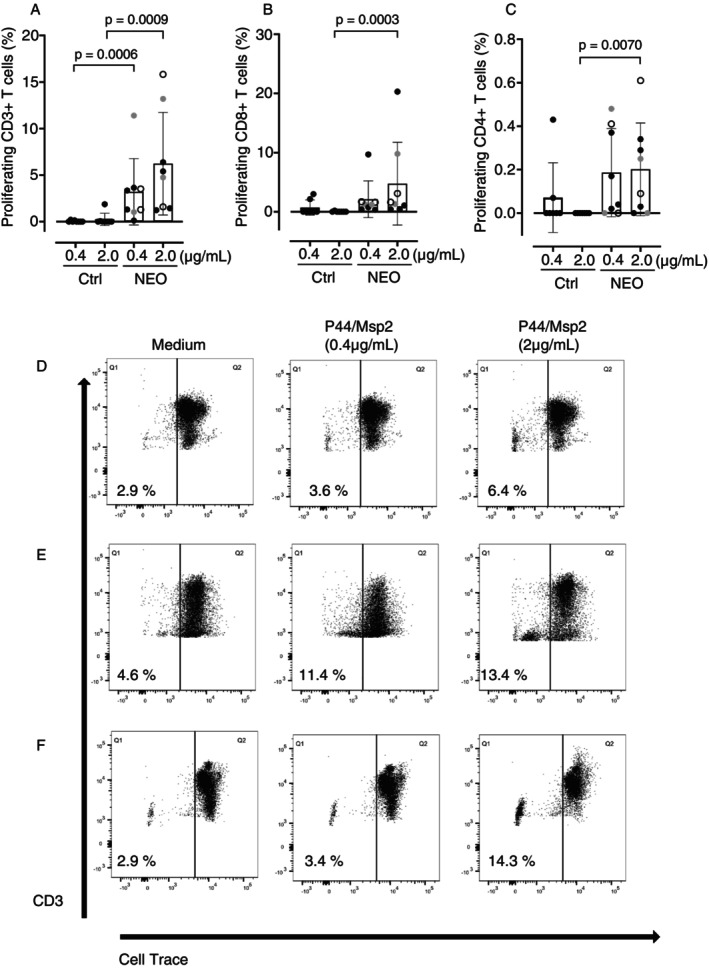
T‐cell proliferation in response to stimulation with P44/Msp2 peptides. The percentages of proliferating (A) CD3+, (B) CD8+ and (C) CD4+ T cells after in vitro stimulation of PBMC with two concentrations of P44/Msp2 peptides for 5 days were measured by assessment of the bleaching of cell‐trace‐stained dividing cells. Lymphoma patients with *Neoehrlichia mikurensis* infection (NEO) (*n* = 7) and matched noninfected lymphoma control patients (Ctrl) (*n* = 7) are shown. Each dot represents one patient; black circles indicate patients who tested positive by *N. mikurensis* PCR before start of rituximab therapy, grey circles denote patients who tested positive by PCR mid‐way, and white circles indicate patients who tested positive by PCR after completion of rituximab therapy. The bars and whiskers indicate means ± SD. Statistical significance was assessed with the Mann–Whitney test. Representative flow cytometry density plots derived from lymphoma patients who tested positive by PCR (D) before, (E) mid‐way and (F) after rituximab therapy. The graphs show the fractions of CD3+ T cells that proliferated after in vitro stimulation with two concentrations of P44/Msp2 peptides for 5 days. Dividing cells gradually lose the cell‐trace stain and shift to the left quadrant of the plot.

*N. mikurensis‐specific T‐cell responses are harboured by lymphoma patients infected with N. mikurensis but not in noninfected lymphoma patients*. Cryopreserved peripheral blood mononuclear cells (PBMC) that were collected before the start of rituximab therapy were tested for T‐cell reactivity to *N. mikurensis* (Study design shown in Figure S1). First, we evaluated the capacity of pooled peptides corresponding to the P44/Msp2 protein of *N. mikurensis* to evoke T‐cell proliferation in the eight patients who were infected with *N. mikurensis*. The CD3+ T cells and CD8+ T cells of *N. mikurensis*‐infected patients proliferated in response to two concentrations of the P44/Msp2 peptides (Figure 1A,B). Only weak proliferation was seen for the CD4+ T cells of these patients in response to the P44/Msp2 peptides (Figure 1C). There was also a dose‐dependent T‐cell proliferative response among the infected patients when comparing the two peptide concentrations (Figure 1A–C). In contrast, low‐level or no T‐cell proliferation was seen among the eight noninfected lymphoma patients (Figure 1A–C), who were selected as the controls for each infected patient by matching for type of lymphoma and sex; age‐matching was possible for only 5/8 patients (Table 2). A representative example of T‐cell proliferation analysed by flow cytometry is shown in Figure 1D. The fraction of CD3+ T cells that proliferated in response to the higher concentration of P44/Msp2 peptides ranged from 1.2% to 17% among the patients with latent infection, whereas this fraction was absent (0%) in seven out of eight matched control patients, except for the control patient in Pair 7, who had 1.9% T‐cell proliferation (Table 3).

**TABLE 2:** Pairing of lymphoma patients infected with *Neoehrlichia mikurensis* with noninfected control lymphoma patients.

Lymphoma patients									
*N. mikurensis* PCR‐positive						*N. mikurensis* PCR‐negative			
Pair No.	Sex	Age	Lymphoma type	Tick bite	Timing of PCR‐positivity^a^	Sex	Age	Lymphoma type	Tick bite
1	M	48	Indolent unclassified lymphoma (splenic)	NR	Before	M	80	Indolent unclassified lymphoma (splenic)	Unaware
2	F	68	Chronic lymphocytic leukaemia	Yes, 2020	Before	F	71	Chronic lymphocytic leukaemia	Yes, 2020
3	M	62	Mantle cell lymphoma	Yes, 2021	Before	M	59	Mantle cell lymphoma	Yes, 2016
4	M	82	Chronic lymphocytic leukaemia	Unaware^b^	Before	M	85	Chronic lymphocytic leukaemia	Unaware
5	M	58	Splenic marginal zone lymphoma	Yes, 2020	Mid‐way	M	88	Splenic marginal zone lymphoma	Unaware
6	M	63	Follicular lymphoma, grade 1–2	Yes, 2022	Mid‐way	M	60	Follicular lymphoma, grade 1	Unaware
7	M	53	Hodgkin's lymphoma, nodular lymphocyte‐dominated	Yes, 2021	After	F	56	Hodgkin's lymphoma, classic with nodular sclerosis + Follicular lymphoma	Unaware
8	F	77	Extra‐nodal marginal zone lymphoma	Yes, 2021	After	M	58	Extra‐nodal marginal zone lymphoma	Unaware

Abbreviations: F, female; M, male; NR, not responded.

^a^ Timing of showing positive in *N. mikurensis* PCR test in relation to the start of rituximab therapy.

^b^ Patient reported being unaware of having been bitten by a tick.

**TABLE 3:** CD3+ T‐cell proliferation in response to stimulation with two concentrations of P44/Msp2 peptides in *Neoehrlichia mikurensis*‐PCR‐positive lymphoma patients (*n* = 8) and matched *N. mikurensis* PCR‐negative lymphoma patients (*n* = 8). The percent proliferation is indicated.

		Pairs of *N. mikurensis*‐infected and noninfected control patients with lymphoma							
P44/Msp2 peptide (μg/mL)	*N. mikurensis* PCR	1	2	3	4	5	6	7	8
0.4	+	3.4	1.3	0.12	3.6	1.0	14	3.5	1.3
0.4	−	0.15	—^a^	0	0	0	0	0.20	0
2.0	+	5.4	1.4	1.2	12	4.7	17	16	1.6
2.0	−	0	—^a^	0	0	0.050	0	1.9	0

^a^ Anergic cells, poor response to mitogen (PHA).

**FIGURE 2:**
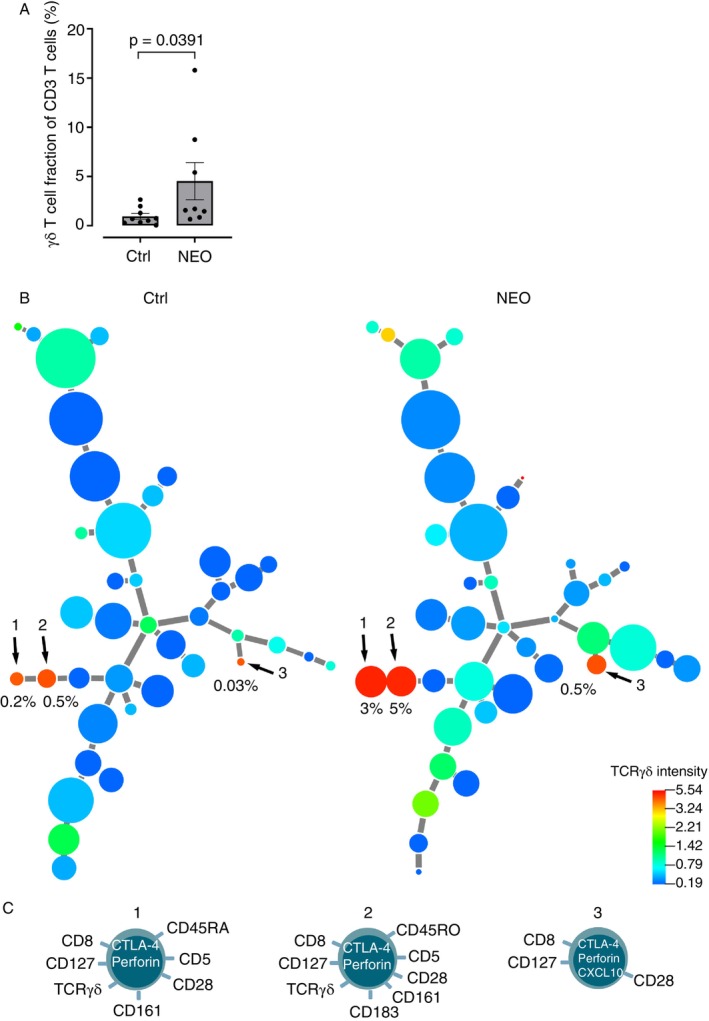
Expanded populations of γδ T cells in lymphoma patients who are infected with *Neoehrlichia mikurensis*. (A) The fractions of γδ T cells among the total population of CD3+ cells were identified in lymphoma patients with *N. mikurensis* infection (NEO) (*n* = 8) and in matched noninfected lymphoma control patients (Ctrl) (*n* = 8) using multiparameter CyTOF analysis. Each dot represents one patient. The bars and whiskers indicate means ± SD. Statistical significance assessed with the Wilcoxon matched‐pairs signed rank test. (B) Minimum spanning tree illustrating γδ‐TCR expression by the CD3+ T cell populations derived from control lymphoma patients without *N. mikurensis* infection (Ctrl; *n* = 8) and from *N. mikurensis*‐infected lymphoma patients (NEO; *n* = 7), as determined using X‐shift clustering analysis. The sizes of the circles represent the average sizes of the T cell populations (*n* = 8 patients). The colour of each circle indicates the magnitude of TCR‐γδ expression, as indicated by the heat map scale. Arrows indicate the T‐cell populations (numbered 1–3) that were expanded in the NEO group, together with the average percentages of the cell populations for the eight patients in each group (Ctrl and NEO groups). (C) Cartoons showing the phenotypes of the γδ T‐cell populations 1, 2 and 3.

*Lymphoma patients with N. mikurensis infection have 10‐fold larger populations of γδ T cells compared with noninfected lymphoma patients*. Multiparameter phenotyping of resting un‐stimulated CD3+ T cells using cytometry by time‐of‐flight (CyTOF) revealed that the infected patients carried a larger population of gamma delta (γδ) T cells than the matched, noninfected control patients with lymphoma (Figure 2A). Cluster analysis disclosed that the eight patients infected with *N. mikurensis* harboured three large populations of γδ T cells, which were also present but significantly smaller in the noninfected lymphoma group of patients (Figure 2B). All three T‐cell populations (named Populations 1–3) were CD8+ and expressed perforin, CD28, IL‐7 receptor (CD127) and cytotoxic T lymphocyte antigen‐4 (CTLA‐4) (Figure 2C). Population 1 differed from Population 2 regarding CD45RA and chemokine receptor CXCR3 (CD183) expression but shared CD161 (killer cell lectin‐like receptor B1) expression, whereas Population 3 was the only one of the three populations that expressed CXCL10, also known as IFN‐γ‐induced protein‐10, IP‐10 (Figure 2C). Moreover, Population 3 was only harboured by one of the eight patients infected with *N. mikurensis*, who conversely lacked Populations 1 and 2 (Pair 6; Table S1). Detailed analysis of the sizes of the three T‐cell populations in the infected (*n* = 8) and noninfected control patients (*n* = 8) revealed that Population 1 was harboured by seven out of eight infected patients and accounted for between 0.5% and 10% of their total CD3+ T‐cell population (average, 3.0%) (Figure 2B and Table S1). In contrast, the same population comprised 0%–0.5% (average, 0.2%) of the CD3+ T cells among the noninfected lymphoma patients. Similarly, the average size of Population 2 was 5.0% of the total CD3+ T‐cell population among the latently infected subjects, but only 0.5% among the noninfected lymphoma patients (Figure 2B). To summarise, Populations 1 and 2 were on average 10‐fold larger in the infected group of patients than in the noninfected group of lymphoma patients.

**FIGURE 3:**
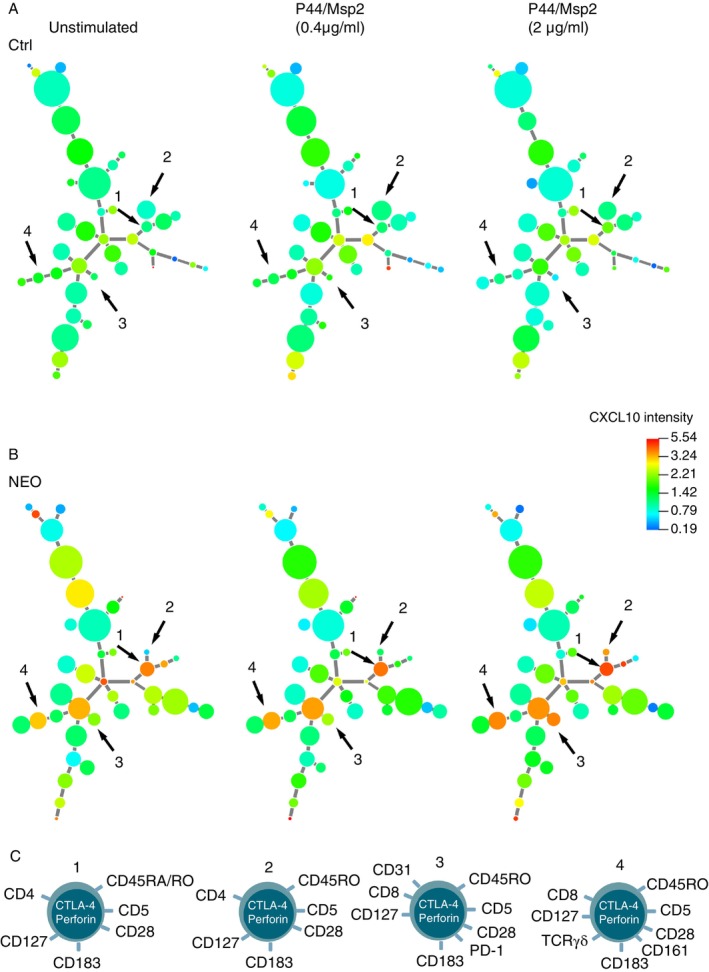
CXCL10 responses triggered by P44/Msp2 stimulation of T cells in vitro. Minimum spanning tree of CXCL10 expression by T‐cell populations derived from: (A) lymphoma patients without *Neoehrlichia mikurensis* infection (Ctrl; *n* = 7); and (B) lymphoma patients with *N. mikurensis* infection (NEO; *n* = 7), as determined by X‐shift clustering analysis. The sizes of the circles represent the average sizes of the T‐cell populations (*n* = 7 patients) after stimulation with two concentrations of P44/Msp2 peptides. The colours of the circles indicate the levels of CXCL10 expression, as shown by the heat‐map scale. Arrows indicate CXCL10‐expressing T‐cell populations that occurred more frequently in the NEO group than in the Ctrl group. (c) Cartoons of the phenotypes of CXCL10‐expressing T‐cell populations 1–4.

**FIGURE 4:**
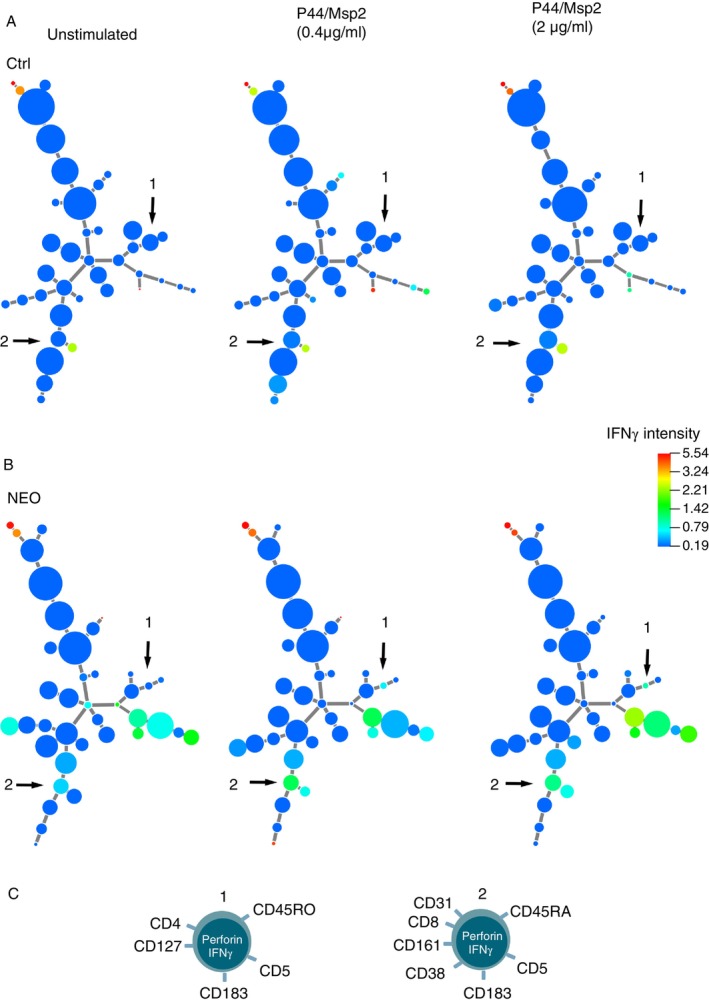
IFN‐γ responses triggered by P44/Msp2 stimulation of T cells. Minimum spanning tree of IFN‐γ‐expressing T‐cell populations derived from (A) noninfected control lymphoma patients (Ctrl; *n* = 7); and (B) lymphoma patients with *Neoehrlichia mikurensis* infection (NEO; *n* = 7), as determined by X‐shift clustering analysis. The sizes of the circles represent the average sizes of the T‐cell populations (*n* = 7 patients) after stimulation with two concentrations of P44/Msp2 peptides. The colours of the circles indicate the levels of IFN‐γ expression, according to the heat‐map scale. Numbered arrows indicate the T‐cell populations that appeared more‐frequently in the NEO group than in the Ctrl group. (C) Cartoons depicting the phenotypes of T‐cell Populations 1 and 2.

*CXCL10 and IFN‐γ responses are evoked* in vitro *by P44/Msp2 stimulation of T cells from patients with N. mikurensis infection*. Cluster analysis also revealed increased expression levels of CXCL10 (Figure 3) and IFN‐γ (Figure 4) by T‐cell populations that were stimulated with P44/Msp2 peptides. There were four T‐cell populations whose CXCL10 expression levels increased dose‐dependently upon in vitro stimulation with the P44/Msp2 peptides in the infected group of patients (Figure 3B); these populations did not respond to peptide stimulation in the noninfected control group of patients (Figure 3A). The T‐cell populations with up‐regulated CXCL10 expression comprised CD4+ Th1 cells (Population 1), effector memory CD4+ Th1 cells (Population 2) and CD8+ effector memory T cells (Populations 3 and 4). All four populations expressed perforin and CTLA‐4.

Two populations of T cells, whose expression of IFN‐γ increased after stimulation with the P44/Msp2 peptides, were identified in the infected group of patients but were absent in the noninfected group of controls (Figure 4A,B). These populations comprised a CD4+ perforin‐expressing Th1 population with weak expression of IFN‐γ (Population 1) and a CD8+ T‐cell population (Population 2) that corresponded to a terminal effector T‐cell population (Figure 4C). Neither CXCL10 nor IFN‐γ was detectable by ELISA in the corresponding cell supernatants of the T‐cell cultures (data not shown).

## 3 Discussion

Numerous intracellular pathogens can persist for extended periods in the human host [19]. Pathogens that reside in immunologically protected niches, of which the vascular endothelium is one example, can give rise to latent infections that are held in control by the immune system. Once the immune defenses are compromised, by aging in the case of *Rickettsia prowazekii* and Brill‐Zinsser disease [20], by tumour necrosis factor (TNF) inhibition in the case of latent TB [21], or by T‐cell suppression in cases of cytomegalovirus reactivation [22], latent infections may reactivate with increased replication of the infectious agent, and if left untreated, they can progress to symptomatic disease. This is in line with our observations for the eight lymphoma patients in the current study, all of whom were unknowingly infected with *N. mikurensis* and lacked the classical signs of neoehrlichiosis, such as fever, pain or vascular events, or any other symptoms or signs that could be attributed to an infectious disease. Our interpretation of the results is that 8% of the study patients had a latent *N. mikurensis* infection and that half of these individuals (4% of the study population) showed evidence of reactivation of a latent infection, namely, undetectable levels of bacteria in the blood prior to the start of rituximab therapy, followed by detectable levels of bacteria in the blood after the start of B‐cell‐suppressive therapy. It seems likely that some, if not all, of the patients would have progressed to symptomatic *N. mikurensis* infection (neoehrlichiosis) if the infection had not been eradicated. This did not happen because all the patients were treated with doxycycline and were PCR‐negative after finishing the course of antibiotics.

We wanted to exclude the possibility that the four patients who putatively underwent reactivation of a latent *N. mikurensis* infection had contracted *de novo* infection through a new tick bite during the study period. To address this question, and to lend support to our theory of latency, we applied the strategy used to diagnose latent TB. IFN‐γ release assays and purified protein derivative (PPD) tests are the gold standards for the diagnosis of latent TB, both of which tests rely on the detection of TB‐specific T‐cell responses, that is, IFN‐γ production by T cells stimulated with TB antigens and the recruitment of T cells to the site of intradermal PPD deposition, respectively [23]. The challenge that we faced was to develop a *N. mikurensis*‐specific T‐cell test, bearing in mind the lack of knowledge regarding the identities of immunogenic proteins of *N. mikurensis*. A further difficulty was that *N. mikurensis* cannot be propagated in cell‐free media, but instead only by culturing on tick cell lines or human endothelial cell lines [8], which limits access to pure bacterial extracts to be used as antigens in T‐cell tests. Consequently, we opted for in silico analysis of the whole‐genome sequences derived from three clinical isolates of *N. mikurensis* that we recently published [4]. The 174‐amino acid P44/Msp2 protein we selected appears to be specific for *N. mikurensis*, as the closest homologue of this protein is harboured by *Ca*. *N. lotoris*, which is found in racoons that do not exist in Europe. We tested a T‐cell stimulation protocol that has been used for studies of T‐cell responses to SARS‐CoV‐2 [24] and found that it was also useful for studies of T‐cell responses to *N. mikurensis*.

The T cells from the eight latently infected lymphoma patients (but not those from the noninfected control patients) responded unequivocally with proliferation and upregulation of CXCL10 and IFN‐γ following stimulation with the P44/Msp2 peptides. It was mainly the CD8+ T cells that proliferated in response to stimulation with the peptides, whereas both CD4+ Th1 and CD8+ T cells up‐regulated the expression of CXCL10 and IFN‐γ. A constant feature was that all the *N. mikurensis*‐responsive T‐cell populations expressed the cytotoxic molecule perforin. Th1 cells and cytotoxic CD8+ T cells are cardinal features of immunity to intracellular pathogens [25], which, as we show in the current study, also applies to human *N. mikurensis* infections. Perforin‐expressing cytotoxic T lymphocytes have been shown to be critical for the clearance of endothelial infection by the intracellular pathogen *Rickettsia conorii* [26]. Th1 cells exert their antimicrobial activities by synthesising IFN‐γ and by providing help to cytotoxic T lymphocytes and B cells for their activation, clonal expansion and, in the case of B cells, antibody synthesis [25, 27, 28]. In fact, antibodies have been shown to participate in the immune defence against several intracellular pathogens through: (i) their capacity to neutralise microbes not only in the extracellular environment but also in the intracellular milieu; and (ii) their ability to facilitate cytotoxic lymphocyte killing of intracellular microbes by way of antibody‐mediated cytotoxicity [25, 27].

Among the latently infected patients, γδ T cells were prominent but did not respond to stimulation with the P44/Msp2 peptides, with one exception: a CXCL10‐expressing γδ T‐cell population (Population 4 in Figure 3C). The nonresponsiveness of γδ T cells to P44/Msp2 peptides was not unexpected since γδ T cells are innate‐like cells that preferentially recognise small, phosphorylated molecules of nonprotein origin and various self‐antigens that are displayed by stressed cells [29, 30], in an MHC‐independent fashion that does not require antigen presentation, as opposed to the classical αβ T cells of adaptive immunity. γδ T cells utilise many different mechanisms to combat intracellular infections, including the secretion of cytolytic perforin and the generation of Th1 cytokines such as IFN‐γ and CXCL10 [31], all of which features appeared among the γδ T‐cell populations described in this study. However, γδ T cells also recognise transformed cells, with a predilection for tumours of B‐cell origin [32], which is highly relevant to our study of patients with malignant B‐cell lymphomas. Nevertheless, the *N. mikurensis*‐infected lymphoma patients had much larger γδ T‐cell populations than the noninfected patients with the same types of lymphoma. This suggests that these expanded γδ T‐cell populations are engendered by the latent *N. mikurensis* infection and are not solely a response to the B‐cell lymphoma.

In summary, the results presented in the current study support the hypothesis that latent *N. mikurensis* infections exist and can undergo reactivation when B‐cell immunity is suppressed. This lends credence to our recently reported finding of a potentially lymphomagenic capacity of *N. mikurensis*, possibly causing B‐cell transformation and lymphoma development in certain patients following a latent *N. mikurensis* infection of long duration [33]. In the future, we aim to develop serologic assays for measuring antibody responses to *N. mikurensis*, which would enable us to define a protective level of anti‐*N. mikurensis* antibodies, below which there lies the risk of an asymptomatic, latent *N. mikurensis* infection becoming symptomatic. Screening of B‐cell lymphoma patients for latent *N. mikurensis* infection prior to the initiation of B‐cell suppressive therapy may also be warranted in *N. mikurensis*‐endemic regions, for example, large parts of Europe and northern Asia [5], to avoid reactivation of these infections and, thereby, avert potentially severe thrombo‐embolic and vascular events.

## 4 Methods

### 4.1 Sex as a Biological Variable

More men than women were recruited to the study, which reflects the higher incidence of lymphoma among men.

### 4.2 Study Design and Participants

Adult patients with malignant B‐cell lymphomas (*n* = 97) who were scheduled to start treatment with rituximab at the Department of Haematology and Coagulation, Sahlgrenska University Hospital, Gothenburg, Sweden, were recruited to the Neoferon study between July 2019 and April 2023. Rituximab was administered either as a single drug or in combination with other immunosuppressive agents and/or chemotherapy. Study participants filled in a questionnaire with items relating to previous tick exposure and tick‐borne diseases, and donated blood on three occasions for the purposes of the study. Blood samples were collected before (24 mL), mid‐way through (10 mL) and after (10 mL) completion of a course of rituximab treatment; the courses typically lasted 4–6 months. PBMC were isolated from heparinised blood collected before the start of rituximab therapy and were cryopreserved at −140°C for subsequent T‐cell analyses. EDTA‐treated blood was used for the *N. mikurensis* PCR. A flow chart of the experimental design of the study is shown in Figure S1.

### 4.3 *N. mikurensis* PCR

Bacterial DNA samples were robot‐extracted (MagNA Pure Compact Extraction Robot; Roche, Basel, Switzerland) from 400 μL of EDTA‐treated plasma (Nucleic Acid Isolation Kit I; Roche) and analysed using a Real‐Time TaqMan PCR that amplified a 169‐bp segment of the *groEL* gene of *N. mikurensis*, as previously described [11]. A synthetic plasmid that contained the 169‐bp sequence cloned into the pUC57 vector (GenScript Biotech, Piscataway, NJ, USA) was used to establish a standard curve. The limit of detection was 1 × 10^3^ bacteria/mL. All positive isolates were confirmed by 16S rRNA‐PCR and sequenced [11].

### 4.4 T‐Cell Proliferation

Within 12 h of blood sampling, PBMC were isolated from heparinised blood using Cytiva Ficoll‐Paque PLUS Media (Fisher Scientific, Hampton, NH, USA) density gradient centrifugation and stored frozen at −140°C until use. Thawed cells were stained with CellTrace Violet (Thermo Fisher, Waltham, MA, USA), suspended in X‐VIVO 15 with gentamicin and phenol red culture medium (Lonza, Basel, Switzerland) and seeded in triplicate (2 × 10^5^ /well) into 96‐well TC‐plates (Sarstedt, Nümbrecht, Germany). The cells were incubated with 41 pooled recombinant peptides (Peptides & Elephants, Hennigsdorf, Germany) (Table S2), covering the entirety of an un‐named *N. mikurensis* outer membrane protein (Genbank; accession no. QXK93293.1) that belongs to the P44/Msp2 family of proteins [4]. Two concentrations, 0.4 and 2.0 μg/mL, of each peptide were used, corresponding to final concentrations of 16 and 82 μg/mL, respectively. The protein sequence encoded in the reference genome *N. mikurensis* SE24 (GenBank accession no. CP066557.1; locus tag HL033_02330; nucleotide positions 577 689–578 213) was used for peptide sequence prediction. Phytohemagglutinin (PHA; Roche) at 5 μg/mL was used as a positive control for T‐cell proliferation, and culture medium was used as a negative control. Cells were harvested after 5 days of culturing, and the supernatants were stored frozen at −80°C for later cytokine analyses. The cells were stained with mouse IgG1 κ antibodies against human CD3 (FITC‐conjugated, clone UCHT‐1), CD4 (APC, clone SK3), CD8 (PE‐Cy7, clone RPA‐T8), CD19 (R718, clone SJ25C1) and CD38 (PE, clone HB7) diluted in FACS buffer (PBS with 2 mM EDTA and 2% FBS), all from BD Bioscience (Franklin Lakes, NJ, USA). The cells were washed, resuspended in 200 μL FACS buffer and stained with 10 μL of 7AAD (BD Bioscience) 10 min before flow cytometry analysis using the FACSLyric instrument (BD Bioscience) equipped with the FACSuite software (BD Bioscience). Data were analysed using the FlowJo ver. 10.7.1 software (Tree Star Inc., Ashland, OR, USA). The gating strategy used for the identification of proliferating CD3+, CD4+ and CD8+ T cells is described in Figure S2A. Proliferation in response to stimulation with P44/Msp2 peptides is expressed as the percent proliferating T cells after subtraction of the spontaneous proliferation in the negative‐control wells.

### 4.5 T‐Cell Populations

PBMC (*n* = 16) were thawed, seeded into 96‐well TC‐plates (2 × 10^5^ cells/well) and incubated with P44/Msp2 peptides for 5 days, as described above. Next, the cells were washed, incubated with 5 μM Cell ID Cisplatin (Fluidigm, South San Francisco, CA, USA), washed and incubated with an antibody panel against 33 extracellular markers for 30 min at room temperature (RT) (Table S3) and 7% Human TruStain FcX (BioLegend, San Diego, CA, USA). The samples were washed, fixed with 1.6% formaldehyde and permeabilised using Foxp3/Transcription Factor Staining buffer (eBioscience, San Diego, CA, USA) for 60 min at RT prior to a 60 min‐incubation at RT with antibodies directed against five intracellular markers (Table S3). The cells were then washed, incubated for 45 min at RT with 62.5 nM Cell‐ID Intercalator‐Ir diluted in Maxpar Fix and Perm Buffer (Fluidigm) and stored at −80°C until analysis using the Helios mass cytometer with the CyTOF ver. 7.0 software (Fluidigm) and gated using the FlowJo 10.8.0 software. The gating strategy is described in Figure S2B. Data are presented as the percentages of cells that expressed the different cell markers. Clustering analysis was performed on gated CD3+ T cells based on 33 channels using the X‐shift algorithm of the VorteX ver. 29/06/17 software. Clustering data are presented using a minimum spanning tree, in which the size of the circle represents the size of the T‐cell population in question and the colour indicates the expression intensity of the selected marker, according to a heat‐map scale. The markers used to define T‐cell subsets are listed in Table S4; effector and memory T‐cell subsets were defined by their expression of CCR7 and CD45 splice variants (CD45RA and CD45RO) [34].

### 4.6 CXCL10 and IFN‐γ ELISA

Quantikine ELISA kits (R&D Systems, Minneapolis, MN, USA) were used for the quantification of CXCL10 and IFN‐γ in cell supernatants using 96‐well Half‐Area Microplates (Corning, Tewksbury, MA, USA).

### 4.7 Statistics

The Wilcoxon matched‐pairs signed rank test was used to compare two groups using the GraphPad Prism ver. 9.0.2 software (GraphPad, San Diego, CA, USA). A *p* < 0.05 was considered statistically significant.

### 4.8 Study Approval

This study was performed according to the ethical guidelines of the Declaration of Helsinki. The study was approved by the Swedish Ethical Review Authority (Dnr. 2020‐00236). All participants provided written informed consent.

## Author Contributions

C.W. conceived the study. C.W., L.W. and C.Li. designed the experiments. L.W. performed the experiments. C.Li. did the cluster analyses. D.J.‐L. performed in silico analyses of the *N. mikurensis* genome. C.Le. provided lymphoma expertise. C.W., L.W. and C.Li. wrote the manuscript, which was revised by all the authors.

## Funding

This work was supported by the Vetenskapsrådet (2020‐01287), ALF (ALFGBG–70150, ALFGBG–966039), the Swedish Cancer Society (21‐1598 Pj), the Cancer and Allergy Foundation (2022‐10671) and the European Union Interreg North Sea Region Program (J‐No. 38‐2‐7‐19).

## Conflicts of Interest

The authors declare no conflicts of interest.

## Supporting information


**Data S1:** imm70120‐sup‐0001‐supinfo.docx.

## Data Availability

The data that support the findings of this study are available from the corresponding author upon reasonable request.
