# Supplementary material for: Latent Neoehrlichia mikurensis Infections May Be Reactivated in Patients With B‐Cell Lymphomas Treated With Rituximab
Source: Immunology. 2026 Feb 5;178(2):307–17. doi: 10.1111/imm.70120 (PMC13135884; doi:10.1111/imm.70120)
Supplement: Supplementary file 1 — Data S1: imm70120‐sup‐0001‐supinfo.docx. [file IMM-178-307-s001.docx]

**Supplemental Table 1.** **Populations of γδ T cells expressed as fractions (%) of the total population of CD3+ T cells in matched pairs of lymphoma patients with and without *N. mikurensis* infection**.

|  | |  | | **Matched Pairs of *N. mikurensis*-infected and non-infected patients** | | | | | | | |
| --- | --- | --- | --- | --- | --- | --- | --- | --- | --- | --- | --- |
| **γδ T-cell population** | ***N. mikurensis* PCR** | | **1** | | **2** | **3** | **4** | **5** | **6** | **7** | **8** |
| **1** | + | | 2.5 | | 3.3 | 0.5 | 1.3 | 1.4 | 0 | 10 | 5.4 |
| **1** | - | | 0 | | 0 | 0 | 0.2 | 0.2 | 0.5 | 0.2 | 0.2 |
|  |  | |  | |  |  |  |  |  |  |  |
| **2** | + | | 1.5 | | 23 | 10 | 0.8 | 1.2 | 0 | 1.6 | 0 |
| **2** | - | | 0.2 | | 0.2 | 0.2 | 0 | 0.2 | 1.2 | 0.4 | 1.2 |
|  |  | |  | |  |  |  |  |  |  |  |
| **3** | + | | 0 | | 0 | 0 | 0 | 0 | 4.1 | 0 | 0 |
| **3** | - | | 0 | | 0 | 0 | 0 | 0 | 0.3 | 0 | 0 |

**Supplemental Table 2.** *N. mikurensis* P44/Msp2 peptide pool.

| **Peptide** | **Name** | **Sequence** |
| --- | --- | --- |
| 1 | p44/Msp2_1 | MAFIPFYSFAQVKGS |
| 2 | p44/Msp2_2 | PFYSFAQVKGSEGSN |
| 3 | p44/Msp2_3 | FAQVKGSEGSNNVNN |
| 4 | p44/Msp2_4 | KGSEGSNNVNNHGFY |
| 5 | p44/Msp2_5 | GSNNVNNHGFYIGGQ |
| 6 | p44/Msp2_6 | VNNHGFYIGGQYKPG |
| 7 | p44/Msp2_7 | GFYIGGQYKPGIGVI |
| 8 | p44/Msp2_8 | GGQYKPGIGVIGDLS |
| 9 | p44/Msp2_9 | KPGIGVIGDLSVKAD |
| 10 | p44/Msp2_10 | GVIGDLSVKADSIDI |
| 11 | p44/Msp2_11 | DLSVKADSIDIKAIL |
| 12 | p44/Msp2_12 | KADSIDIKAILALKA |
| 13 | p44/Msp2_13 | IDIKAILALKADAAA |
| 14 | p44/Msp2_14 | AILALKADAAAENPA |
| 15 | p44/Msp2_15 | LKADAAAENPAGANN |
| 16 | p44/Msp2_16 | AAAENPAGANNVPSE |
| 17 | p44/Msp2_17 | NPAGANNVPSEVSKF |
| 18 | p44/Msp2_18 | ANNVPSEVSKFIQKP |
| 19 | p44/Msp2_19 | PSEVSKFIQKPDNFK |
| 20 | p44/Msp2_20 | SKFIQKPDNFKGFYK |
| 21 | p44/Msp2_21 | QKPDNFKGFYKPTYN |
| 22 | p44/Msp2_22 | NFKGFYKPTYNNSFA |
| 23 | p44/Msp2_23 | FYKPTYNNSFAGFSG |
| 24 | p44/Msp2_24 | TYNNSFAGFSGLIGY |
| 25 | p44/Msp2_25 | SFAGFSGLIGYSTPN |
| 26 | p44/Msp2_26 | FSGLIGYSTPNGVRL |
| 27 | p44/Msp2_27 | IGYSTPNGVRLELEG |
| 28 | p44/Msp2_28 | TPNGVRLELEGSFEN |
| 29 | p44/Msp2_29 | VRLELEGSFENFELK |
| 30 | p44/Msp2_30 | LEGSFENFELKNSNK |
| 31 | p44/Msp2_31 | FENFELKNSNKCTLK |
| 32 | p44/Msp2_32 | ELKNSNKCTLKNAYK |
| 33 | p44/Msp2_33 | SNKCTLKNAYKYFAA |
| 34 | p44/Msp2_34 | TLKNAYKYFAAAAKL |
| 35 | p44/Msp2_35 | AYKYFAAAAKLKANN |
| 36 | p44/Msp2_36 | FAAAAKLKANNDEID |
| 37 | p44/Msp2_37 | AKLKANNDEIDDAAA |
| 38 | p44/Msp2_38 | ANNDEIDDAAAGENE |
| 39 | p44/Msp2_39 | EIDDAAAGENENHNK |
| 40 | p44/Msp2_40 | AAAGENENHNKYLII |
| 41 | p44/Msp2_41 | GENENHNKYLIIIMF |

**Supplemental Table 3.** Antibody and elemental isotope tags for CyTOF.

| **No.** | **Specificity*** | **Metal label**** | **Clone** |
| --- | --- | --- | --- |
| 1 | CD45 | Y89 | HI30 |
| 2 | CD107a | Cd106 | H4A3 |
| 3 | CD69 | Cd113 | FN50 |
| 4 | **IFN-γ** | Cd116 | B27 |
| 5 | CD196/CCR6 | Pr141 | G034E3 |
| 6 | CD19 | Nd142 | HIB19 |
| 7 | CD5 | Nd143 | UCHT2 |
| 8 | CD31 | Nd144 | WM59 |
| 9 | CD4 | Nd145 | RPA-T4 |
| 10 | CD8 | Nd146 | RPA-T8 |
| 11 | CD20 | Sm147 | 2H7 |
| 12 | CD274/PD-L1 | Nd148 | 29E.2A3 |
| 13 | CD25 | Sm149 | 2A3 |
| 14 | CD134/OX40 | Nd150 | ACT35 |
| 15 | CD123/IL-3R | Eu151 | 6H6 |
| 16 | TCRγδ | Sm152 | 11F2 |
| 17 | CD185/CXCR5 | Eu153 | RF8B2 |
| 18 | CD3 | Sm154 | UCHT1 |
| 19 | CD45RA | Gd155 | HI100 |
| 20 | CD14 | Gd156 | HCD14 |
| 21 | CD194/CCR4 | Gd158 | L291H4 |
| 22 | CD161 | Tb159 | HP-3G10 |
| 23 | CD28 | Gd160 | CD28.2 |
| 24 | **Ki-67** | Dy161 | B56 |
| 25 | CD66b | Dy162 | 80H3 |
| 26 | CD183/CXCR3 | Dy163 | G025H7 |
| 27 | CD45RO | Dy164 | UCHL1 |
| 28 | CD279/PD-1 | Ho165 | EH12.2H7 |
| 29 | CD197/CCR7 | Er167 | G043H7 |
| 30 | CD127/IL-7Ra | Er168 | A019D5 |
| 31 | CD278/ICOS | Tm169 | C398.4A |
| 32 | **CD152/CTLA-4** | Er170 | 14D3 |
| 33 | CD38 | Yb172 | HIT2 |
| 34 | HLA-DR | Tb173 | L243 |
| 35 | **CXCL10***** | Yb174 | J034D6 |
| 36 | **Perforin** | Lu175 | B-D48 |
| 37 | CD56 | Yb176 | NCAM16.2 |
|  | Cell-ID Cisplatin | Pt195 | - |
| 38 | CD16 | Bi209 | 3G8 |
|  | DNA1 | 191Ir | - |
|  | DNA2 | 193Ir | - |

*Intracellular markers are indicated in bold type.

** All metal-labelled antibodies except for the antibody against CXCL10 were purchased

from AH Diagnostics, Aarhus, Denmark.

*** In-house conjugated antibody.

**Supplemental Table 4.** Markers used to define the T-cell subsets.

| **T-cell subset** | **Markers** |
| --- | --- |
| Th1 | CD3+/CD4+/CXCR5-/CXC3+/CCR6- |
| Th2 | CD3+/CD4+/CXCR5-/CXC3-/CCR6- |
| Th17 | CD3+/CD4+/CXCR5-/CXC3-/CCR6+ |
| Th follicular | CD3+/CD4+/CXCR5+ |
| CTL | CD3+/CD8+ |
| Naïve  Terminal effector | CD3+/CCR7+/CD45RA+/CD45RO-  CD3+/CCR7-/CD45RA+/CD45RO- |
| Effector memory | CD3+/CCR7-/CD45RA-/CD45RO+ |
| Central memory | CD3+/CCR7+/CD45RA-/CD45RO+ |
| Activated T cells | CD3+/HLA-DR+ |

Th, T helper; CTL, cytotoxic T lymphocyte.

**Supplemental Figure 1.**

**SFigure 1 Experimental study design**. All study patients (*n* = 97) were tested for *N. mikurensis* by PCR on three occasions during the course of rituximab therapy. Sixteen patients, 8 of whom tested positive during the study period and 8 matched patients with the same type of lymphoma who remained negative for *N. mikurensis* during the entire study period, were evaluated for *N. mikurensis*-specific T cells before the start of rituximab therapy (time 0). Patients were treated with doxycycline for 3 weeks immediately after they tested positive for *N. mikurensis* by PCR.

**Supplemental Figure 2.**


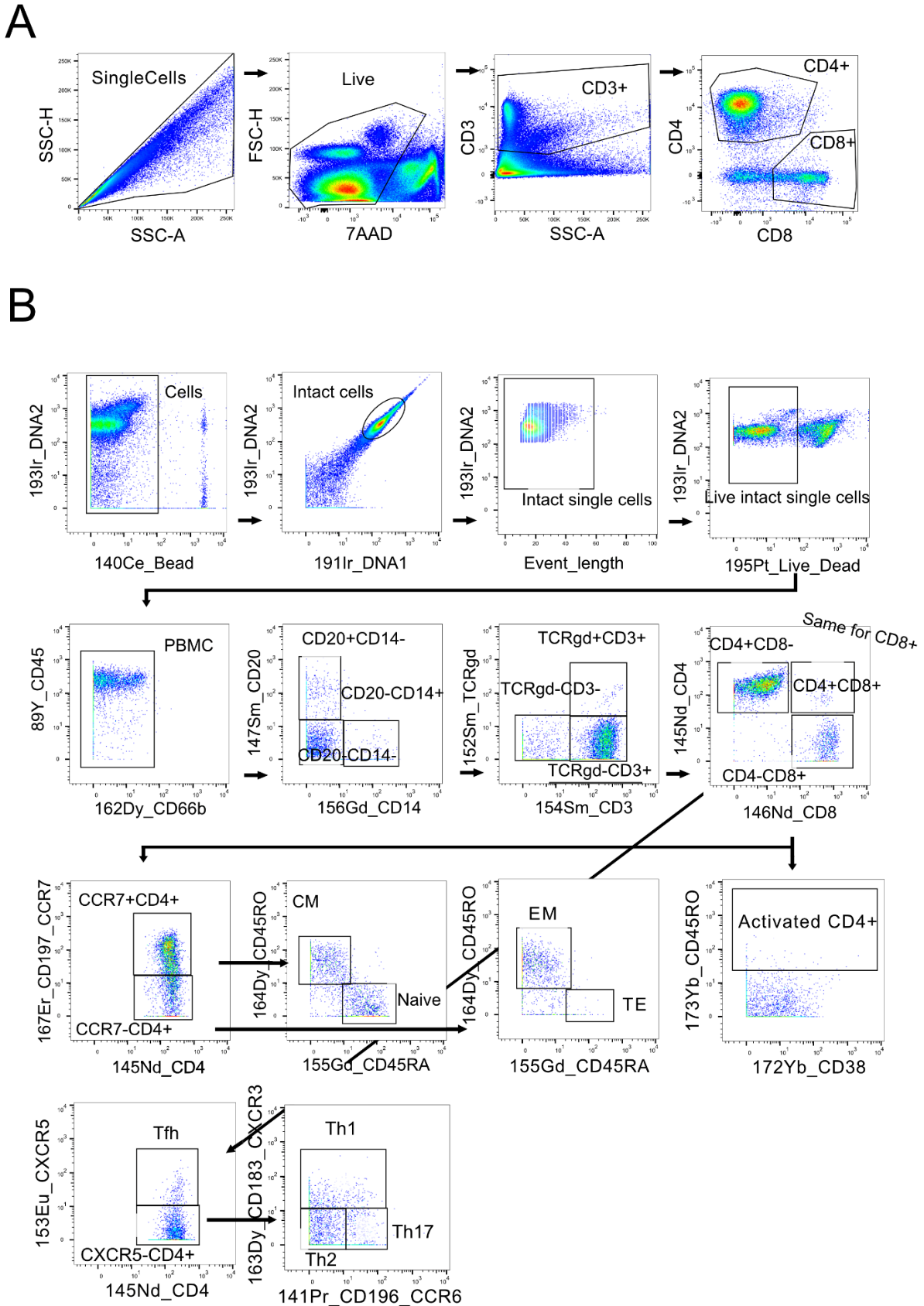


**SFigure 2 Gating strategy for samples analysed with flow cytometry and CyTOF**. **(A)** CD4+ T cells and CD8+ T cells were gated by flow cytometry from single live cells with high-level expression of CD3. (**B**). The gating of samples analysed with CyTOF was performed on intact, single live cells, followed by the gating of lymphocytes, NK cells, monocytes, and dendritic cells. CD4+ and CD8+ T cells were gated from TCR γδ-negative CD3+ T cells. The third and fourth rows show the gating strategy used to identify central memory (CM), naïve, effector memory (EM), terminal effector (TE), and activated CD4+ T cells; the same procedure was used for CD8+ T cells. T-follicular helper cells (Tfh) were gated based on the expression of CD185/CXCR5 and CD4. The gating of Th1, Th2 and Th17 cells is based on CD185/CXCR5- cells, followed by the expression of CD183/CXCR3 and CD196/CCR6.
